# ﻿A new species of *Hibiscus* (Malvaceae, Malvoideae) from Guyana

**DOI:** 10.3897/phytokeys.214.91586

**Published:** 2022-11-22

**Authors:** Laurence J. Dorr

**Affiliations:** 1 Department of Botany, MRC-166, National Museum of Natural History, Smithsonian Institution, P.O. Box 37012, Washington, D.C. 20013-7012, USA Department of Botany, MRC-166, National Museum of Natural History, Smithsonian Institution Washington United States of America

**Keywords:** Guyana, *Hibiscus*, Malvaceae, Malvoideae

## Abstract

*Hibiscusmarioniae* Dorr, **sp. nov.** is described and illustrated. It evidently is restricted to central Guyana, northeast of the Kanuku Mountains near the Rewa River, a tributary of the Rupununi River. The new species is most similar morphologically to *H.amazonicus* Fryxell, which was described from Amazonas, Brazil.

## ﻿Introduction

A collection of *Hibiscus* L. (Malvaceae, Malvoideae) made by Marion J. Jansen-Jacobs in central Guyana northeast of the Kanuku Mountains near the Rewa River in 1999 was identified by the late Paul A. Fryxell as “Hibiscusaff.verbasciformis Klotzsch ex Hochr. vel sp. nov.” No additional material of this taxon has been collected since then, nor have additional specimens been found in searches of herbaria (CAY, K, Naturalis, NY, P, US, W) with rich collections of specimens from the Guianas. Likewise, nothing that matches the Jansen-Jacobs collection has been reported from Brazil (Fernandes Júnior and Coutinho 2022) or the adjacent Venezuelan Guyana (Fryxell 2001). This Guyanese collection does not match *H.verbasciformis*, which is an illegitimate superfluous name for *H.spathulatus* Garcke, as closely as it matches *H.amazonicus* Fryxell. In French Guiana especially, *H.amazonicus* has been confused with and misidentified as *H.spathulatus*. Although similar to *H.amazonicus*, the *Hibiscus* collection made by Jansen-Jacobs in Guyana is distinct and is described and illustrated below.

## ﻿Taxonomic treatment

### ﻿ Hibiscus marioniae

Taxon classificationPlantaeMalvalesMalvaceae

Dorr sp. nov.

9A2A71E0-1637-59F3-B289-1EA5185BF015

urn:lsid:ipni.org:names:77308754-1

Fig. 1

#### Diagnosis.

*Hibiscusmarioniae* Dorr differs from *H.amazonicus* Fryxell in having elliptic (versus ovate) leaf blades with cuneate (versus cordate to truncate and often asymmetrical) bases, a crenulate (versus coarsely toothed) margin, and long acuminate (versus acute) apices; more numerous (12 versus 8) and narrower (ca. 1.0 versus (2.0–)3.0–6.0 mm broad) involucellar bracts that are very slightly spathulate (versus distinctly spathulate, broadly lanceolate or imperfectly stipitate-peltate); and glabrous (versus minutely scaberulous) seeds.

**Figure 1.**
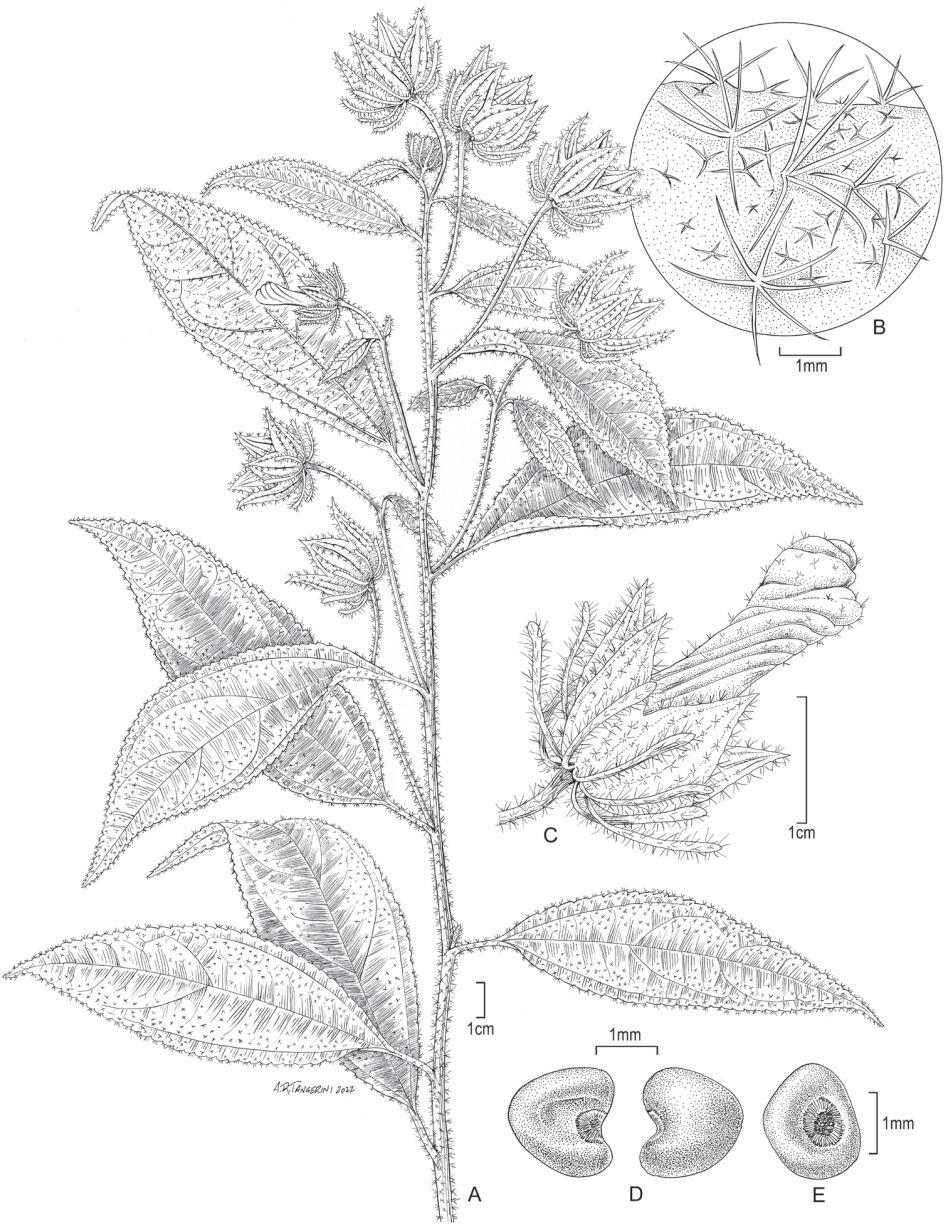
*Hibiscusmarioniae* Dorr, sp. nov. **A** habit **B** detail of calyx showing vestiture **C** flower showing corolla, calyx, and involucellar bracts **D** seeds lateral views **E** seed showing hilum (**A–E** from *Jansen-Jacobs et al. 6011*).

#### Type.

Guyana. [Upper Takutu-Upper Essequibo]: Upper Essequibo Region, Rewa River, Spider Mountains, 03°08'N, 058°32'W, 400–500 m alt., 20 Sep 1999 (fl, fr), *M.J. Jansen-Jacobs, B.J.H. ter Welle, P.P. Haripersaud, O. Muller & M. van der Zee 6011* (holotype: U barcode 0067247!; isotypes: NY!, TEX barcode 00568796 as image!).

#### Description.

Suffrutescent herbs, to 50 cm tall; stems woody at base, unarmed, sparingly to moderately pubescent, with appressed 4–8-armed stellate hairs ca. 1 mm in diameter. Leaves simple, elliptic, 8.5–13.0 × 2.5–4.0 cm, base cuneate, palmately 3-nerved at base, midrib and 2° nerves slightly raised above, prominent below, margin crenulate, apex long acuminate, leaf blades sparingly pubescent above and below with yellowish, bifurcate and stellate hairs, stellate hairs 4–8-armed, arms ca. 1.0 mm long, erect, bifurcate hairs more frequent below than above; petioles 1.5–2.5 cm long, with a ventral line of short whitish stellate-hairs and more conspicuous and abundant yellowish stellate hairs, the latter hairs not in a line and denser distally; stipules almost linear, ca. 1.0–2.0 mm long, caducous. Flowers solitary or paired in leaf axils toward apices of stems; pedicels 3.5–7.0 cm long, not articulated, pubescent with ± appressed stellate hairs and more conspicuous, 4–8-armed stellate hairs with arms to 2.0 mm long. Bracts of involucel 12, distinct, 10.0–15.0 × ca. 0.75–1.0 mm, not or scarcely exceeding united portion of calyx at anthesis, very slightly spathulate apically, plane, with simple, bifurcate, and stellate hairs, hairs or arms of stellate hairs to 2.0 mm long. Calyx 5-lobed, united ca. half way, lobes 1.0–1.2 × 0.5–0.6 cm at anthesis, broadly triangular, apices acute, papery, light green with 3 darker but not thickened veins, nectaries absent, outer surface with yellowish simple and mostly 4-armed stellate hairs, arms to 2.0 mm long, inner surface with a few scattered simple hairs, accrescent in fruit, turning blackish (on herbarium specimens, at least), lobes expanding to 2.0–2.5 × 1.2 cm. Petals ca. 2.5 cm long (only one flower seen and not dissected), white (fide *Jansen-Jacobs et al. 6011*), corolla shape unknown. Staminal column shorter than the petals; anthers purple (fide *Jansen-Jacobs et al. 6011*). Styles and stigmas not seen. Capsules enclosed in accrescent calyces, 5-locular, chartaceous, capsule walls undulate and molded around individual seeds, walls covered with minute whitish hairs and more conspicuous yellowish simple hairs to 2.0 mm long. Seeds ca. 2.0 × 2.0 mm, globose-reniform, brownish (the hilum blackish), glabrous.

#### Etymology.

The species epithet honors Marion J. Jansen-Jacobs who has contributed greatly to our understanding of the flora of the Guianas as collector, herbarium curator, author, and executive director of the Flora of the Guianas project.

#### Distribution and ecology.

Known only from the type collection, which was made in central Guyana, northeast of the Kanuku Mountains; 400–500 m alt. According to information on the specimen label, the plant was found in an open spot in forest on rock in the “Spider Mountains.” The name of these mountains does not appear in standard gazetteers (Stephens and Traylor 1985; Anonymous 1993; Guyana Lands and Surveys Commission 2019) and it may have been a name created by the collectors of the type.

#### Discussion.

*Hibiscus* as traditionally treated is a species-rich genus of ca. 200 (Hochreutiner 1900) to over 400 species (POWO 2022). It is included in the Hibisceae, a tribe defined by loculicidally dehiscent (“capsular”) fruit, lack of gossypol glands, 5-toothed staminal column apex, styles usually apically branching, stigmas usually terminal, and style branches equal in number to the carpels (Pfeil et al. 2002). Molecular data, however, suggest that the traditional concept of *Hibiscus* that is based on morphology created a “severely” paraphyletic genus (Pfeil et al. 2002; Pfeil and Crisp 2005; Koopman and Baum 2008), which has other tribes (Decaschistieae and Malvavisceae) and other genera of Hibisceae nested within it. Infrageneric classification of *Hibiscus* also has been problematic (Pfeil and Crisp 2005) with unresolved conflicts between the sectional classifications based on morphology proposed by de Candolle (1824), Grisebach (1859), Gürke (1892), Hochreutiner (1900), Ulbrich (1921), van Borssum Waalkes (1966), and Fryxell (1988). This makes assigning *H.marioniae* to a section problematic.

Morphologically, *Hibiscusmarioniae* is most similar to *H.amazonicus*. The two species share unarmed stems, an involucel comprised of distinct bracts, a papery or chartaceous 5-fid calyx that is ca. half-divided and accrescent in fruit, and a staminal column shorter than the petals. When Fryxell (1984) described *H.amazonicus*, he compared it to three other species found in South America (*H.dimidiatus* Schrank, *H.sororius* L., and *H.spathulatus* Garcke) and stated they formed a natural grouping or alliance, but he refrained from naming this group. It appears that Fryxell made the connection to this group because as stated in his protologue, *H.amazonicus* keyed out (somewhat ambiguously) in Gürke (1892) and Kearney (1957) either to *H.sororius* or *H.verbasciformis* (≡ *H.spathulatus*). Morphologically, especially in vestiture, these three species are very similar to each other, and they only can be distinguished easily by the shape of their involucellar bracts.

Earlier, Grisebach (1859) had made *Hibiscussororius*, which also occurs in the West Indies and Mexico, the type and sole member of H.sect.TrionastrumGrisb. He categorized this section as having distinct involucellar bracts that are apically enlarged, a 5-fid ventricose calyx, and glabrous seeds. While *H.amazonicus*, *H.marioniae*, and *H.spathulatus* could be placed in this section, the cordate-ovate involucellar bracts of the closely allied *H.dimidiatus* and the puberulous or hirtellous seeds of *H.spathulatus* and *H.dimidiatus*, respectively, would require modification of Grisebach’s circumscription of his monotypic section.

Gürke (1892) overlooked Hibiscussect.Trionastrum when he revised the Brazilian species of *Hibiscus* and he placed *H.sororius*, *H.dimidiatus*, and *H.spathulatus* in sect.Ketmia (Mill.) DC. (≡ sect.Hibiscus), which he defined by involucellar bracts subulate-falciform, setaceous, linear, lanceolate, ovate, spathulate, or dilated apically but not bifurcate, and calyx not inflated with eglandular lobes. These characters, especially the negative or absent ones, seem to have been selected to set this section apart from sect.Furcaria DC. Nonetheless, little seems to separate sect.Trionastrum from sect.Hibiscus apart from the calyx being ventricose (i.e., swollen or distended) in the former and not inflated in the latter. Also, the seeds of sect.Hibiscus are either glabrous or pubescent but without the distinctive cotton-like hairs found in sect.Bombicella. Fryxell (1988) later recognized both sect.Hibiscus and sect.Trionastrum in his treatment of the Mexican species of *Hibiscus* and placed *H.sororius* in sect.Trionastrum but said nothing about the other South American allies of this species, presumably because they do not occur in Mexico.

Hochreutiner (1900) in his revision of *Hibiscus* had a very different assessment of the relationships of the three species that Fryxell (1984) later thought to be allied with *H.amazonicus*. Hochreutiner (1900), who also overlooked H.sect.Trionastrum, placed *H.sororius* in sect.Spatula Hochr., which he defined by its apically dilated involucellar bracts and glabrous seeds; *H.dimidiatus* in sect.Trichospermum Hochr. (= sect.Hibiscus); and *H.spathulatus* (as *H.verbasciformis*) in sect.Trionum DC. He clearly was unhappy with his sectional placement of *H.spathulatus* since he acknowledged that it was the only species that he included in sect.Trionum with lanceolate bracts and deeply lobed calyces.

No members of Hibiscussect.Trionastrum (or sect.Spatula) were included in the analysis of Pfeil and Crisp (2005: table 2). However, given that *H.marioniae* and its presumed relatives have distinct involucellar bracts and non-inflated calyces, these species will probably be found to belong to the “/Euhibiscus” clade, a rank free clade recognized by Pfeil and Crisp (2005) that contains sect.Hibiscus. Thus, irrespective of how the paraphyly in *Hibiscus* and the Hibisceae eventually is resolved taxonomically, *H.marioniae* likely will remain in or closely allied to *Hibiscus* s.str.

## Supplementary Material

XML Treatment for Hibiscus marioniae
